# Faba bean populations already contain the inbreds needed for breeding

**DOI:** 10.1007/s00122-026-05259-w

**Published:** 2026-06-12

**Authors:** Henri Laugel, Wolfgang Link

**Affiliations:** Department of Crop Sciences, Division of Plant Breeding Methodology, University of Goettingen, Carl-Sprengel-Weg 1, 37075 Goettingen, Germany

## Abstract

**Key message:**

Highly inbred individuals are naturally present in faba bean populations and should be used for rapid and cost-efficient development of line and synthetic cultivars.

**Abstract:**

Producing inbred lines in faba bean (*Vicia faba* L.) breeding is, although crucial, a long, costly and laborious process due to the partial allogamy of the crop. However, in any genetically diverse and open pollinating faba bean population, this particular reproduction system leads to the so-called identity disequilibrium, resulting in a significant proportion of individuals being highly inbred. This study presents a cost-efficient approach to identify these highly inbred individuals from populations with a limited number of KASP markers. Using simulations, the most accurate inbreeding coefficient estimator from limited number of markers (ten to 100) was found to be the so-called estimator F_L_ (which is based on a maximum likelihood approach). In the “Göttingen Winter Bean Population”, 8.4% of individuals (61 out of 728) were classified as highly inbred (F_L_ ≥ $0.8\overline{3 }$). We evaluated the performance of classifying highly inbred individuals using a limited number of markers with both simulated and empirical data, allowing to identify strategies for optimizing this approach. Using 50 markers and a threshold of F_L_ ≥ 0.9, we identified 42 individuals in our population, including 36 truly highly inbred individuals (out of 61) and six insufficiently inbred individuals (false positives). Furthermore, applying a preliminary step with a low number of markers to eliminate insufficiently inbred individuals successfully permitted a marked reduction in genotyping cost (up to 46%). Faba bean breeders can greatly benefit from the presented approach by identifying highly inbred individuals fast, at low cost and with limited labour, even though no DH technology is available.

**Supplementary Information:**

The online version contains supplementary material available at 10.1007/s00122-026-05259-w.

## Introduction

Inbred lines are crucial for faba bean breeding. Highly homozygous inbred lines are “immortal” and stable over generations when propagated through selfing. They are used as line cultivars or as components of population cultivars. One major challenge in faba bean breeding is the development of such inbred lines. It is a lengthy, laborious and costly process, especially due to the lack of an efficient double haploid (DH) technique in faba bean (Hale et al. 2022). Non-inbred individuals must be propagated through generations of selfings to reach the level of inbreeding necessary to be considered as inbred line. Because of its partial allogamous mode of reproduction (Brünjes & Link 2025; Duc et al. 2010; Link et al. 1994), self-fertilization of faba bean is carried out in isolation cages to ensure absence of (cross-)pollinating insects (honeybees and bumblebees). In addition to this special effort, the low and varying auto-fertility rate (Brünjes & Link Brünjes and Link, 2025) of many genotypes requires manual, mechanical stimulation (moving the petals; the so-called tripping) to improve seed set through selfing.

This particular mode of reproduction of faba bean makes line development tedious, costly and slow. But the same particular reproductive mode can be exploited to find inbred individuals that are already available; instead of producing them the tedious way. Highly inbred individuals indeed can be found in faba bean populations. Population genetics tells that any genetically diverse and open pollinating (i.e. with access of pollinators) faba bean population expectedly consists of cohorts of individuals with differing inbreeding status (inbreeding cohorts). Each such inbreeding cohort contains individuals that share the same number of successive, uninterrupted generations of self-fertilization in its pedigree. This genetic composition of partially allogamous populations was presented by Cockerham and Weir (1984) and Kelly and Williamson (2000), and specifically in faba bean by Link et al. (1994) and Brünjes and Link (2021). Assuming a self-fertilization rate of 0.5 (a realistic value), each individual in a population has, for example, a 0.5^3^ probability to stem from three consecutive generations of self-fertilization. Cross-fertilization rates within faba bean populations are typically intermediate, reportedly never near 0% or 100% in Europe or Canada (Gasim et al. 2004, 2011, 2002; Holden & Bond 1960; Marzinzig et al. 2018; McVetty & Nugent-Rigby 1984; Metz et al. 1992, 1995; Poulsen 1975; Suso et al. 2005, 2006; Suso & Maalouf 2010). Faba bean also exhibits marked yet limited inbreeding depression, which is clearly demonstrated by many successful line varieties (Abdelmula et al. 1999; Adhikari et al. 2021; Bishnoi & Panchta 2012; Bond 1982; Ebmeyer 1988; Fleck & Ruckenbauer 1989; Gallais 1992; Ghaouti 2007; Wright 1973, 1977; Zeid et al. 2004). Partial allogamy generates highly inbred individuals, which are part of the population and its dynamic. Therefore, we expected to find a certain proportion of such individuals.

So far, these highly valuable individuals were not used in breeding, and this was only because they are phenotypically not recognizable. They can be easily detected by estimating the inbreeding coefficient (F) of individuals using molecular markers. Multiple genotyping techniques are available: notably genotyping-by-sequencing (GBS), specific primer enrichment technology (SPET), and SNP arrays (O’Sullivan et al. 2019). However, these techniques require a certain budget. To genotype with the “Vfaba_v2” Axiom SNP array costed between 50 and 100€ per individual (in 2023). Individual F estimates can be obtained inexpensively with a limited number of competitive allele-specific PCR (KASP) markers. KASP analyses costed less than 0.40€ per individual per marker (in 2024).

In this study, our aim was to develop a method to identify and hence select the a priori available highly inbred individuals from faba bean populations using a limited number of KASP markers, to reduce the time, labour and cost of the inbred line development process. Our objectives were to (1) define which individuals are sufficiently inbred to be used as source of inbred line in breeding, (2) assess the accuracy of several F estimators with a limited number of markers, (3) quantify the frequency of highly inbred individuals (their incidence) in existing faba bean populations applying the most accurate estimator, (4) analyse in detail the performance of the classification of individuals as highly inbred with limited number of markers, and (5) calculate the genotyping cost reduction achieved through the use of a preliminary classification step (using an even smaller number of markers).

## Materials and methods

### Definition of highly inbred individuals

In classical faba bean breeding, inbred line cultivars are defined as being derived from at least F4 individuals (Adhikari et al. 2021; personal communication with faba bean breeders), i.e. derived from at least three consecutive generations of self-fertilization after the Mendelian generation F1. Highly inbred individuals could be defined as those which belong to the inbreeding cohort F4 or higher (F5, F6…). However, the pedigree of the individuals in a population is not known. There is quantitative variation in F values between individuals within each inbreeding cohort. We investigated whether individuals can be unambiguously classified into inbreeding cohorts based on genotypic data, taking this variation into account.

We modelled the distribution of F values of faba bean individuals per inbreeding cohort, utilizing the work of Hillel et al. (1990) about the distribution of parental genomes in selfing and backcrossing. The average F of F4 individuals is 0.875 and varies quantitatively depending on the number of independently segregating genomic segments of the relevant species. A segment is characterized as a chromosome or a portion of a chromosome that is enclosed by crossing-over sites. To determine the number of such genomic segments in faba bean (which has 2n = 12 chromosomes), we used the genetic map built from NV644 (Kasztelan) x NV153 (IG12658) using 6607 SNPs (Webb et al. 2016) and found an expected number of 24 such segments. We defined the distribution of F values per inbreeding cohort using the binomial distribution:

1 $$P\left({F}_{Fx}=\frac{{N-N}_{AB}}{N}\right)=\left(\frac{\left(\frac{N}{{2}^{x-1}}\right)!}{{N}_{AB}!\left(\frac{N}{{2}^{x-1}}-{N}_{AB}\right)!}\right){\left(\frac{1}{2}\right)}^{\left(\frac{N}{{2}^{x-1}}\right)},$$

where F_Fx_ is the F of individuals from the inbreeding cohort Fx, where (x-1) is the number of successive generations of self-fertilization the individuals are derived from (for example, individuals of the F2 cohort originate from one generation of self-fertilization), N the number of independently segregating genomic segments (in our case, N = 24; when $\frac{N}{{2}^{x-1}}$ is not an integer, the result is rounded to the nearest integer), and N_AB_ the number of homologous segments originating from different gametes, considering the F1 hybrid from which the individuals are derived is constituted of gamete A and B.

We followed the simplification in Hillel et al. (1990) and considered the genomic segments to be of same size and constant across generations. The F distribution of an inbreeding cohort (Fx) was constructed from an “average” genome of the preceding cohort (Fx-1). For example, we constructed the F distribution of F4 individuals by self-fertilizing the “average” genome of a F3 individual (F = 0.75), instead of accepting an accumulation of variation for F already starting in generation F2 and F3. The deficit of variance for F values among F4 individuals caused by this simplification depends on the number of segments and is negligible for most species (Hillel et al. 1990).

**Fig. 1:**
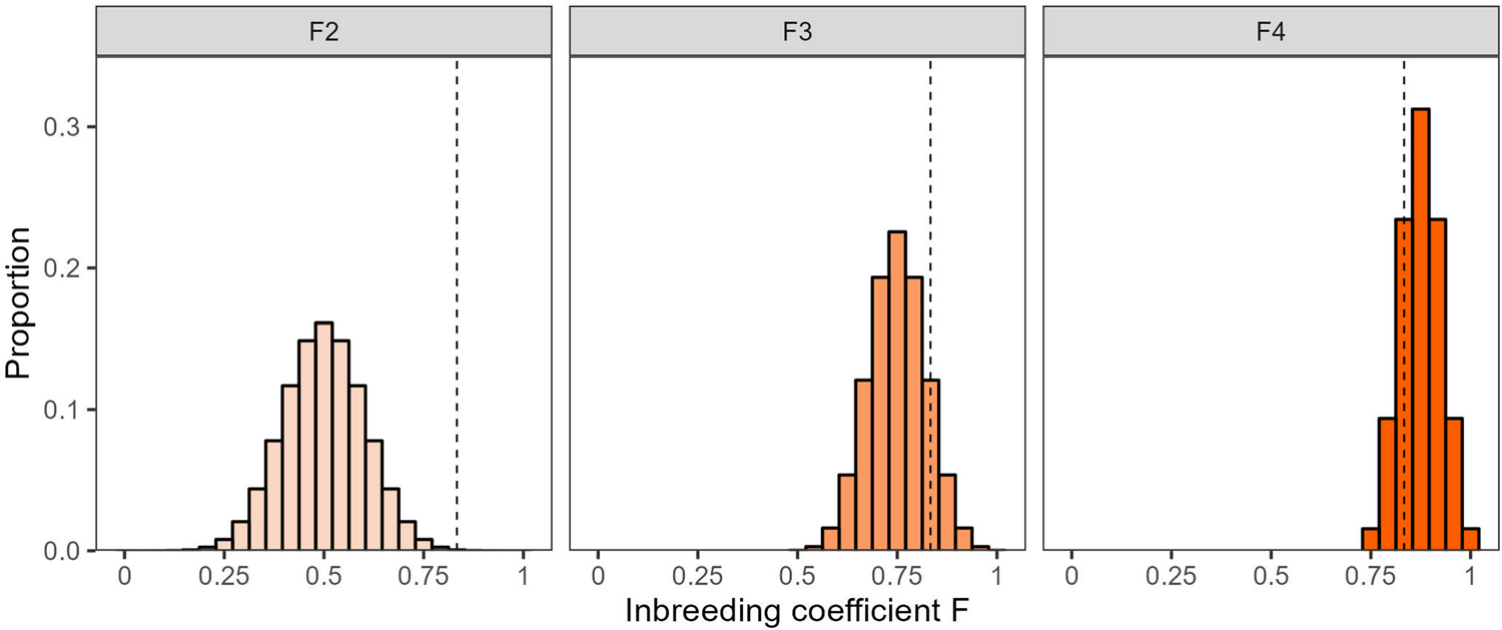
Simulated F distribution of faba bean individuals belonging to the inbreeding cohort F2, F3 and F4. The dashed vertical line corresponds to $0.8\overline{3 }$, which is the 25% quantile of the distribution of F4 individuals

Our theoretical quantitative variation in F values per inbreeding cohort leads to an overlap in the distribution of individual F values between cohorts (Fig. 1). It is not possible to unambiguously determine whether a faba bean individual with F = 0.75 is a F2, F3 or F4 individual. Yet, to qualify an individual as highly inbred, we are not bound to the number of consecutive selfings such as 3 or 4 or 5, but to its realized F. We defined these individuals as highly inbred, if their F value is equivalent to that of individuals belonging to the inbreeding cohort F4 or higher. We arbitrarily decided to set our so-called inbreeding threshold to $0.8\overline{3 }$, corresponding to the 25% quantile of the F distribution of individuals from the F4 inbreeding cohort (Fig. 1). Individuals with a F value greater or equal to this threshold were defined as “highly inbred”, while those lower to it were defined as “insufficiently inbred”. This way, we considered most of the F4 individuals and all individuals of the higher inbreeding cohorts as highly inbred. Few F3 individuals were also included as highly inbred, those which by chance already went beyond our inbreeding threshold (F = $0.8\overline{3 }$) with only two successive generations of selfings.

The distribution of F2 individuals (Fig. 1) fitted quite accurately the empirical distribution observed by Schön (1993) from 380 F2 maize individuals. The latter shows slightly smaller variance, which is expected from a species like maize with a higher number of chromosomes (2n = 20).

### Genotypic data from the GWBP

The Göttingen Winter Bean Population (GWBP) is a famous academic germplasm. It was initiated in 1989 by equally mixing 11 founder inbred lines: six from Germany (Webo, Wibo, Hiverna/1, 79/79, L977/88, and L979/S1), two from France (Côte d’Or/1 and Arrisot), and three from the UK (Banner, Bourdon, and Bulldog). The mixture was grown in open pollinated conditions, with access of pollinating bees and bumble bees, and experienced no artificial and limited natural selection. Since then, this population was maintained and continued in Göttingen each season, the current generation in 2025 is its 37th generation. Hence, the GWBP is a freely recombining partially allogamous population, presumably near to its breeding system equilibrium (Gasim 2003; Gasim et al. 2004; Link & Arbaoui 2006). It is the essential germplasm of winter faba bean breeding in Germany (Link & Arbaoui 2006).

We sampled six 10-mm-diameter leaf discs from 728 random individual plants from the population and acquired their genotypic data using the “Vfaba_v2” Axiom SNP array, containing approximatively 60 k markers (O’Sullivan et al. 2019). DNA extraction and genotyping analyses were performed by SGS – INSTITUT FRESENIUS GmbH, TraitGenetics Section in Gatersleben. The 728 individuals originated from two generations of the GWBP: 318 individuals were obtained from seeds harvested in 2020, and 410 from seeds harvested in 2022. At these two generations, the population should have reached equilibrium by far. Between 2020 and 2022, we do not expect change due to drift nor selection, since number of individuals was always about 1000 and practically no winter kill occurred. This assumption is corroborated by very little difference in allele frequencies between the two subsets, no noteworthy difference observed comparing their respective distribution of homozygosity rate and no evidence of population structure through principal component analysis (PCA) and admixture analysis (using the “LEA” R package; v.3.22.0). We obtained a fixation index (FST) of 0.001 between the two subsets (using the “hierfstat” R package; v.0.5—11), fully supporting our assumption. Hence, we considered the 728 individuals as one sample from one same population.

Markers with less than 10% missing values were selected, and markers with a major allele frequency p > 0.99 were eliminated. The markers were filtered based on Fisher’s linear discriminant (FLD), measuring the cluster quality of a SNP. The genotypic data were obtained through three distinct chip analyses, resulting in three FLD values. We selected the markers which had an average FLD value greater or equal to seven. It resulted in a total of 11,467 markers.

### Data simulation

**Fig. 2:**
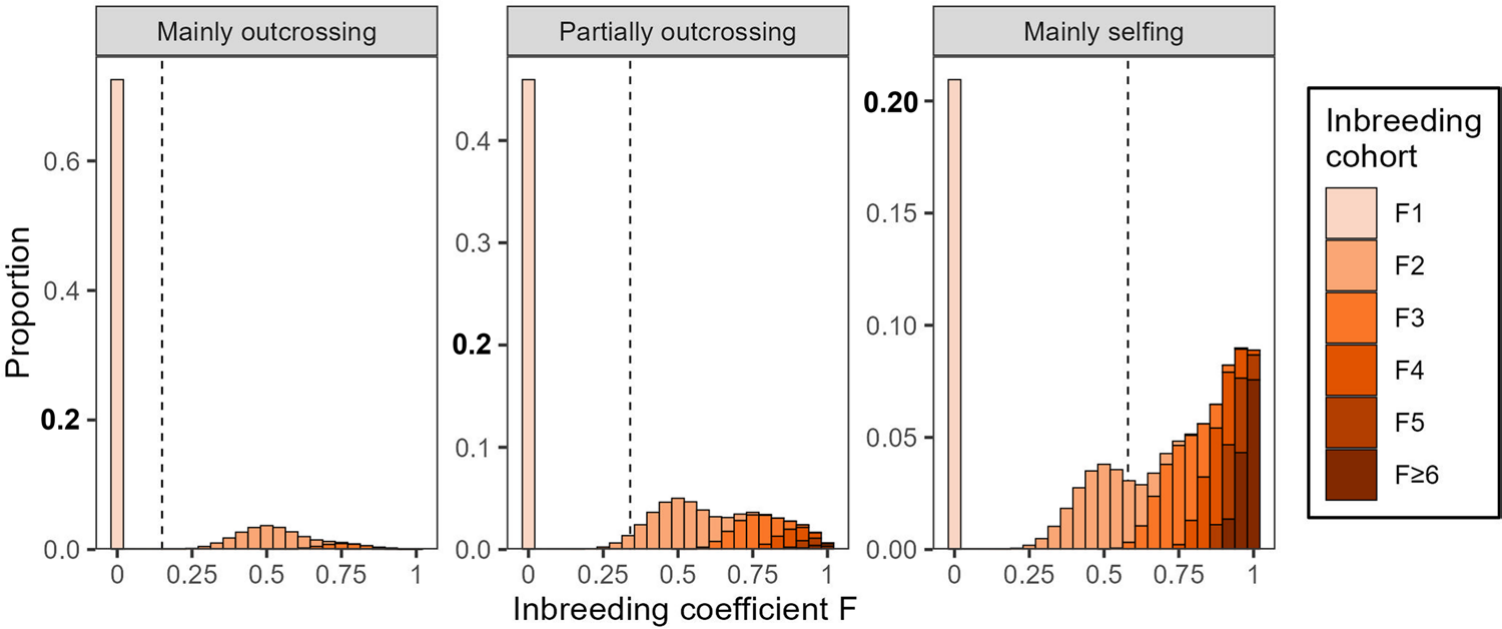
Stacked histogram representing the proportion of individual F values for three simulated populations according to the inbreeding cohorts (colours): from left to right, the mainly outcrossing population ($\overline{\mathrm{S} }$ = 0.27), the partially outcrossing population ($\overline{\mathrm{S} }$ = 0.53) and the mainly selfing population ($\overline{\mathrm{S} }$ = 0.78). The dashed vertical lines represent the mean F of each population

We simulated in R the distribution of individual F of three equilibrium populations with differing degree of outcrossing (Supplementary material 1; Fig. 2) defined in this study as: (1) the mainly outcrossing population, (2) the partially outcrossing population and (3) the mainly selfing population.

Ordered by descending amount of outcrossing rate, we observed respectively a population mean F of 0.15, 0.34 and 0.60, and a population mean self-fertilization rate ($\overline{\mathrm{S} }$) of 0.27, 0.53 and 0.78. The proportion of highly inbred individuals according to our inbreeding threshold (F ≥ $0.8\overline{3 }$), was, respectively, 1.3%, 10.6% and 38.2%.

### Marker subsets

To assess the accuracy of the F estimation and the performance of the classification of highly inbred individuals from such faba bean populations, we generated F estimates from marker subsets. We generated marker subsets with marker number from five to 100, by increment of 5; and we generated such marker subsets with either random allele frequencies or with ideal allele frequencies. “Ideal” means *p* = 0.5 throughout, no missing values and zero linkage disequilibrium (LD). Such a balanced allele frequency makes marker most informative on the quantification of the identical-by-descent (IBD) share of homozygosity, if such share was added to the alike-in-state (AIS) share of homozygosity. Zero LD ensures that two markers contribute independent information on the inbreeding status of the individual.

For empirical data from the GWBP, random marker subsets were generated by initially picking 100 random markers from our 11,467 markers. These markers were then used to generate progressively smaller sets through sequential five-marker reductions, resulting in subsets ranging from 100 markers down to five markers. Each smaller set was derived from its next-larger set. We repeated this to obtain 50 different marker subsets for each marker number.

From our 11,467 markers, we selected markers with major allele frequency p $\in$[0.50, 0.55], less than 5% missing values, and r^2^ of LD lower than 0.2, resulting in a total of 372 markers. Marker pairs showing LD of r^2^ > 0.1 were representing 1.4% of the latter near-to-ideal marker set, against 13.6% of the entire 11,467-marker set. We repeated the same procedure as described above to generate the ideal marker subsets: 100 markers were picked from the 372 at random, and the other marker subsets were derived from it. The procedure was iterated 50 times to obtain 50 different ideal marker subsets for each marker number.

For simulated data, we generated 50 subpopulations of 728 individuals with individual F values drawn from the three theoretical distributions of individual F (Fig. 2), to mimic our approach with empirical data. For each subpopulation, we initially generated a marker subset of 100 markers, and simulated genotypic data in R for all individuals, using their assigned F values and the major allele frequencies of the markers. For “random” marker subsets, the markers had allele frequencies randomly drawn from a uniform distribution (0.50 to 0.99). For “ideal” marker subsets, all the markers had *p* = 0.5. We derived progressively smaller subsets through sequential five-marker reductions.

### Inbreeding coefficient estimation

The accuracy of several F estimators as depending on number of markers used (five to 100) was evaluated by simulated data in R. Accuracy was assessed through the root mean square error (RMSE), calculated based on the deviation of the 728 estimated F values from their corresponding true F value. We studied the RMSE obtained solely from the partially outcrossing population, as its self-fertilization rate ($\overline{\mathrm{S} }$ = 0.53) was most realistic and closely aligned with the value expected in our GWBP, and also in other material reported in the literature (Brünjes & Link 2021). Caballero et al. (2022) reviewed several methods to estimate the within-population inbreeding coefficient F of individuals with biallelic marker data of which several were used in this study (Table 1).

**Table 1:** List of six F estimators to estimate F from a limited number of markers with their respective formula presented under a common notation (see their respective notation in the referenced papers in Supplementary material 2). In the formulas, m is the total number of markers, x_i_ the number of minor alleles at locus i, and p_i_ the major allele frequency at locus i

Estimator	Formula	References
F_LH1_	${F}_{LH1}=1- \frac{\sum_{i=1}^{m}{x}_{i}(2-{x}_{i})}{\sum_{i=1}^{m}2{p}_{i}(1-{p}_{i})}$	Li & Horvitz 1953
F_LH2_	${F}_{LH2}=1-\frac{1}{m}\sum_{i=1}^{m}\frac{{x}_{i}(2-{x}_{i})}{2{p}_{i}(1-{p}_{i})}$	Caballero et al. 2022
F_VR1_	${F}_{VR1}=\frac{\sum_{i=1}^{m}{({x}_{i}-2{p}_{i})}^{2}}{\sum_{i=1}^{m}2{p}_{i}(1-{p}_{i})}-1$	VanRaden 2008
F_VR2_	${F}_{VR2}=\frac{1}{m}\sum_{i=1}^{m}\left(\frac{{({x}_{i}-2{p}_{i})}^{2}}{2{p}_{i}(1-{p}_{i})}-1\right)$	VanRaden 2008
F_YA1_	${F}_{YA1}=\frac{\sum_{i=1}^{m}{{x}_{i}}^{2}-\left(1+2{p}_{i}\right){x}_{i}+{{2p}_{i}}^{2}}{\sum_{i=1}^{m}2{p}_{i}(1-{p}_{i})}$	Zhang et al. 2022
F_YA2_	${F}_{YA2}=\frac{1}{m}\sum_{i=1}^{m}\frac{{{x}_{i}}^{2}-\left(1+2{p}_{i}\right){x}_{i}+{{2p}_{i}}^{2}}{2{p}_{i}(1-{p}_{i})}$	Yang et al. 2010

In addition, we evaluated the so-called likelihood estimator F_L_, described in detail in Supplementary material 3 and exemplified in Supplementary material 4.

When the allele frequency of all markers is *p* = 0.5, the algebras of all presented estimators collapse to the same equation:

2 $$F=1-\frac{2 }{m}\sum_{i=1}^{m}{x}_{i}\left({2-x}_{i}\right)=1-\frac{2H}{m},$$

where m is the total number of polymorphic markers, and H the number of observed heterozygous loci.

Within simulated ideal subsets, all markers have no missing value, zero LD, and *p* = 0.5. With such ideal marker subsets, the accuracy of all F estimators (Table 1 and F_L_) is identical. Therefore, we investigated the accuracy of the F estimators solely with simulated random marker subsets.

F values must range from 0 to 1, but estimates of F can take values out of this range due to sampling error. We constrained the estimated F values from 0 to 1 for all estimators. Any estimates below 0 were set to 0, while values above 1 were set to 1.

#### Homozygosity rates

We defined individuals as highly inbred based on their F. Yet, it is genuinely their genome-wide homozygosity rate that matters when these individuals are used as source of inbred lines. The amount of residual segregation in a not-fully inbred line is caused by genetic loci which were “still” heterozygous in the ancestral individual of the line.

We checked whether F estimators were more accurate than homozygosity rates (HOM) to estimate true genome-wide homozygosity rates. In our simulation, considering an infinite number of loci, the true F of the individuals were perfectly correlated with the true genome-wide homozygosity rate. So, the RMSE of estimated homozygosity rates (HOM) relative to the true genome-wide homozygosity rates could be directly compared to the RMSE of F estimates. The true genome-wide homozygosity rate of the simulated non-inbred individuals (F = 0) was deduced based on the uniform distribution (from 0.50 to 0.99) and was found to be around 0.659. To deduce the true genome-wide homozygosity rate of inbred individuals, we projected a linear relationship between true F and true genome-wide homozygosity rate:

3 $$H=0.659+F*\left(1-0.659\right),$$

where H is the true genome-wide homozygosity rate of an individual, and F the true F value of this same individual.

### Presence and proportion of highly inbred individuals in existing faba bean populations

To check the presence and quantify the proportion of highly inbred individuals within faba bean populations, we used the most accurate F estimator. We estimated the individual F values using allele frequencies derived from the population sample, making our sample the reference population (Wang 2022). We first investigated the distribution of individual F values of the GWBP using the 728 individuals, genotyped with the Axiom SNP array (11,467 filtered markers). We deduced the mean self-fertilization rate of the GWBP based on the following equation:

4 $$\overline{S }=\frac{2\overline{F} }{\overline{F }+1},$$

where $\overline{\mathrm{S} }$ is the mean self-fertilization rate of the population and $\overline{\mathrm{F} }$ the mean F of the population. The equation assumes S to be constant over generations (Kempthorne 1957; Li & Horvitz 1953; Wright 1921).

To check the findings from our Axiom chip genotyped, main set of 728 individuals, we genotyped 624 additional random individuals from the GWBP using 50 near-to-ideal markers to estimate their F values. The mean self-fertilization rate of this supplementary sample of the GWBP was also deduced based on Eq. (4).

To corroborate findings from this single population (GWBP), we in addition investigated the proportion of highly inbred individuals within two generations (Syn-4 and Syn-8) of a candidate synthetic cultivar (spring faba bean), in collaboration with the breeding company NPZ Hans-Georg Lembke KG. This population was composed of four highly inbred components. A total of 456 individuals of the population were analysed in generation Syn-4 and 347 individuals of its generation Syn-8. All 803 individuals were genotyped with 43 KASP markers that differed for their allele frequencies. Since in Syn-4 and probably even in Syn-8, equilibrium was not reached, we deduced the population $\overline{\mathrm{S} }$ based on the following equation:

5 $${F}_{t}=\frac{s}{2-s}\left[1-{\left(\frac{s}{2}\right)}^{t}\right]+ {(\frac{s}{2})}^{t}*{F}_{0},$$

where ${\mathrm{F}}_{\mathrm{t}}$ is the mean population F at generation t, s the mean population S, t the current generation of the population, and ${\mathrm{F}}_{0}$ the mean F of the initial population. The equation assumes s to be constant over generations (Kempthorne 1957).

### Classification of highly inbred individuals

We wanted to classify individuals from faba bean populations as either insufficiently inbred or highly inbred. Individuals were defined as truly insufficiently inbred or highly inbred using (1) the inbreeding threshold (F = $0.8\overline{3 }$). In empirical data, the truly highly inbred individuals (total positives) and truly insufficiently inbred inbred individuals (total negatives) were defined based on F values obtained from the 11,467-marker set using the most accurate F estimator. Based on estimated F values (from limited marker data), the inbreeding threshold (F = $0.8\overline{3 }$) was not directly applied. Instead, individuals were classified as highly inbred using (2) the so-called inbreeding classification threshold (ICT). With limited marker numbers, individuals with an estimated F value greater or equal to that ICT (F ≥ ICT) were classified as “highly inbred”, while those lower to it (F < ICT) were classified as “insufficiently inbred”.

To assess the performance of our classification, we studied:

(1) The number of true positives (TP): number of correctly classified truly highly inbred individuals,

(2) The number of false positives (FP): number of truly insufficiently inbred individuals wrongly classified as highly inbred.

An effective classification resulted in a high number of TP and a low number of FP. A classification was most effective when resulting in all truly highly inbred (total positives) classified as TP and zero FP.

We examined the effects of (1) the population composition, (2) the accuracy of F estimation and (3) the classification threshold (ICT) on the numbers of TP and FP. We used simulated data to investigate the influence of these three factors on the classification performance. For (1) the population composition, we compared the results obtained from the three simulated populations (the mainly outcrossing, the partially outcrossing and the mainly selfing populations; Fig. 2). The influence of (2) the F estimation accuracy was investigated by varying the number of markers used (five to 100) and their allele frequency (random or ideal). We report the number of TP and FP based on (3) four ICT values: $0.8\overline{3 }$, 0.90, 0.95 and 1.00. We avoided ICT < $0.8\overline{3 }$, because in that case, the classification produced FP, even with maximum F estimation accuracy, which went against our intention.

To study the transferability of our simulated results, we assessed the classification performance using our empirical data from the GWBP and compared the results with those of the simulated partially outcrossing population ($\overline{\mathrm{S} }$ = 0.53).

### The preliminary classification step

We tested the use of a preliminary classification step, meant to save genotyping cost. This step aimed to eliminate most of the insufficiently inbred individuals with a low number of markers in a first step (cheap). The DNA samples and the genotypic data (from these few markers) of the non-eliminated individuals are reused for the next step. These remaining individuals are fewer and thus less costly to analyse with more markers (second step). Individuals are ultimately classified as highly inbred in this second, main classification step.

We tested this preliminary classification step with our empirical data. We coined the term preliminary inbreeding classification threshold (PICT) as the ICT used in the preliminary classification step. Its role was to eliminate as many truly insufficiently inbred individuals, while keeping all truly highly inbred ones. We permissively set this PICT as the ICT value correctly classifying all TP with 95% confidence (the 5% quantile of the per-iteration maximal ICT values that classified all TP correctly across the 50 marker subsets). It was defined using simulations from our simulated partially allogamous population and was specific for the number of markers used for F estimation and their random or ideal allele frequency. These PICT values were then applied to our empirical data, and the performance of the preliminary classification step was investigated (looking on the number of insufficiently inbred and highly inbred individuals eliminated).

To assess the cost efficiency of using the preliminary classification step, we compared the cost of the approach with and without it, according to the number of markers used in both steps. The cost was not calculated in euro, but as a multiplier to the cost of KASP analysis per individual per marker, named KASP cost factor, excluding the cost of KASP assay setup and DNA extraction (which are constant whether using the preliminary classification step or not):

6 $$KASP cost factor=N*{M}_{1}+\left({TP}_{1}+{FP}_{1}\right)*\left({M}_{2}-{M}_{1}\right),$$

where N is the total number of analysed individuals from the population, M_1_ the marker number used in the preliminary classification step (for the one-step approach, M_1_ = 0), M_2_ the marker number used in the main classification step, TP_1_ the number of true positives in the preliminary classification step, and FP_1_ the number of false positives in the preliminary classification step (for the one-step approach, TP_1_ + FP_1_ = N).

## Results

### Inbreeding coefficient estimation

**Fig. 3:**
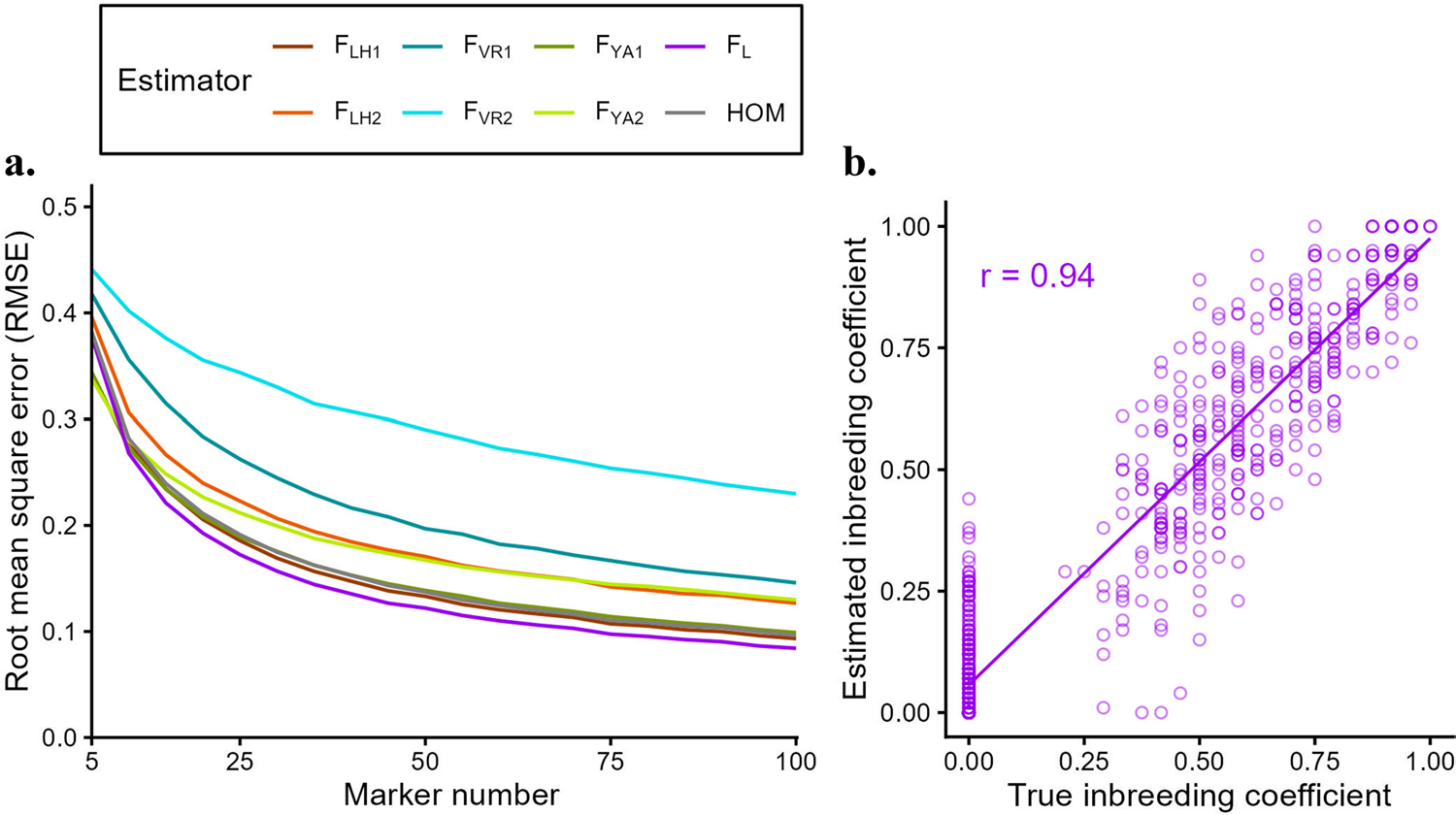
**a** Root mean square error (RMSE) of the tested F estimators in the simulated partially outcrossing population according to the number of markers used for F estimation with random allele frequencies. The lines represent the mean across the 50 marker subsets. **b** Scatter plot of estimated F values of 728 individuals from the simulated partially outcrossing population, obtained from 50 random markers using the likelihood estimator F_L_, against their true F values

With random marker subsets, we observed differences in accuracy between the F estimators (Fig. 3a). As expected, the accuracy of the F estimation increased with the number of markers used. The improvement was greatest between five and 25 markers. For the methods of Li and Horvitz (F_LH_), VanRaden (F_VR_) and Yang et al. (F_YA_), the first version of the method (F_LH1_, F_VR1_ and F_YA1_) had greater accuracy than their respective second version (F_LH2_, F_VR2_ and F_YA2_; Table 1). The likelihood estimator F_L_ was the most accurate for all marker numbers employed (except with five markers). When at least 20 markers were used, the estimator F_LH1_ was the second most accurate estimator. Yet, to reach the accuracy of the estimator F_L_ using 50 markers, 60 markers were needed using the estimator F_LH1_. Using 50 markers, the Pearson correlation between estimated F_L_ values and true F values was 0.94 on average (Fig. 3b). This correlation decreased to 0.81 when excluding individuals with a true F value of 0.

The accuracy of the HOM estimates was not as good as the one obtained from the most accurate F estimators (F_LH1_ and F_L_), but it was reasonably high, outperforming all other F estimators with 50 random markers, for example. Its RMSE line is slightly above the one from the F_LH1_ estimator, nearly overlapping (Fig. 3a). HOM estimates may be useful, for example if the allele frequencies of the markers are inaccurate, because estimated from a very limited number of individuals.

### Presence and proportion of highly inbred individuals in existing faba bean populations

**Fig. 4:**
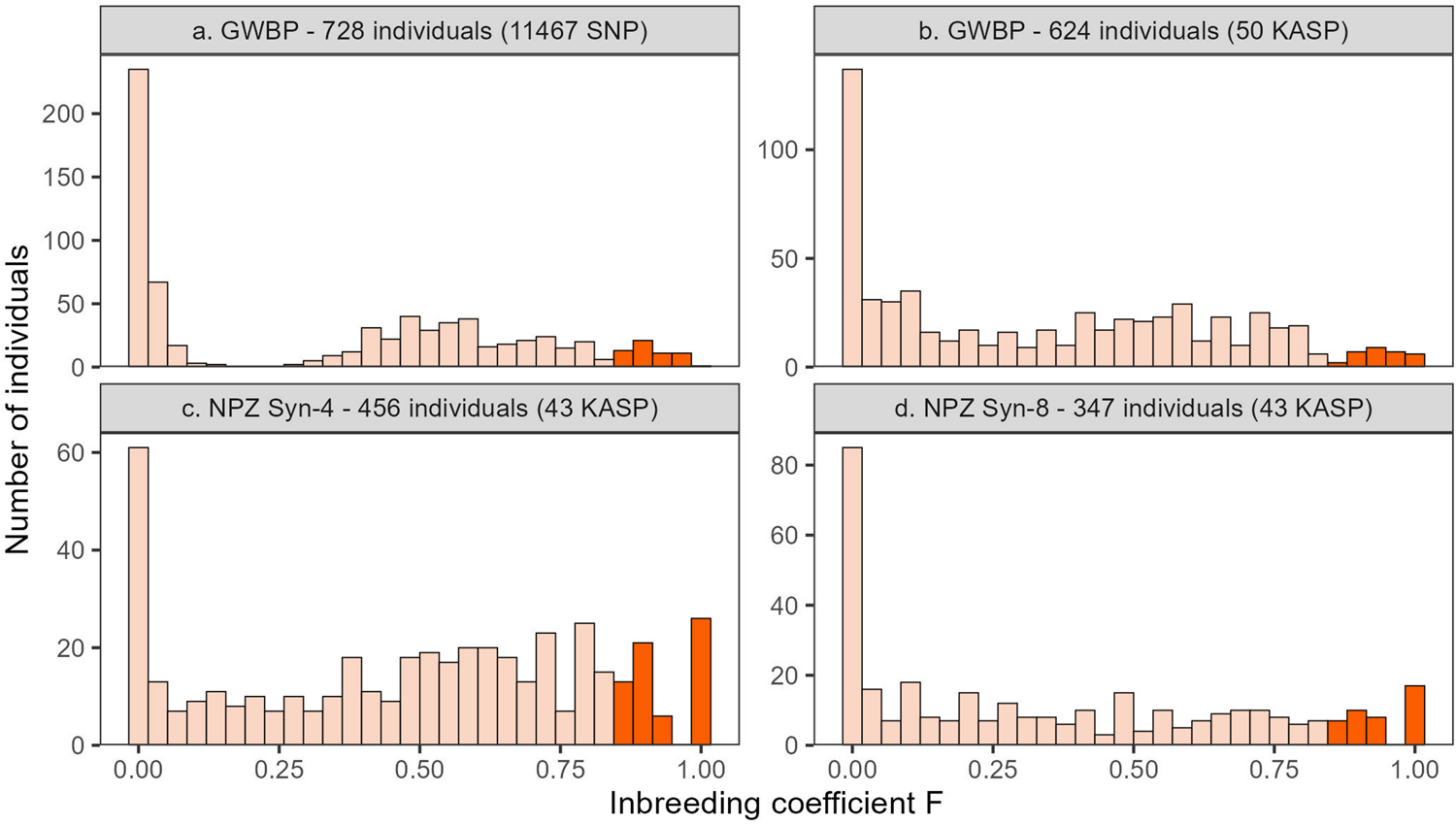
Distribution of estimated individual F values in the Göttingen Winter Bean Population (GWBP) of **a** individuals genotyped using the Axiom SNP array (11,467 SNP markers) **b** individuals genotyped using 50 near-to-ideal (p≈0.5; LD≈0) KASP SNP markers and in the candidate synthetic cultivar from the breeding company NPZ Hans-Georg Lembke KG in **c** Syn-4 and **d** Syn-8 generations. Highlighted in darker orange is the proportion of highly inbred individuals, based on our inbreeding threshold (F ≥ $0.8\overline{3 }$)

Using the most accurate estimator (F_L_), we estimated the distribution of individual F values of (1) the 728 individuals from the GWBP genotyped with the Axiom SNP array (11,467 markers; Fig. 4a), (2) the supplementary 624 individuals from the GWBP genotyped with the 50 near-to-ideal KASP markers (Fig. 4b) and (3) the two generations of the candidate synthetic spring bean cultivar (Syn-4 and Syn-8) genotyped with 43 KASP markers (Fig. 4c and d).

The F_L_ values of the individuals genotyped with the Axion SNP array from the GWBP (Fig. 4a) show the same type of bimodal distribution previously observed with the simulated F distribution of the partially outcrossing population (Fig. 2). Since the F estimates were bounded to F ≥ 0, the average F of non-inbred individuals is slightly biased towards positive F values. Their proportion is slightly lower than in the simulated partially outcrossing population (0.42 < 0.45). A total of 61 out of 728 individuals (about 8.4% of the entire population) were estimated to have a F greater or equal to our inbreeding threshold (F_L_ ≥ $0.8\overline{3 }$) and were thus considered as highly inbred. This proportion was slightly lower compared to the simulated partially outcrossing population (8.4% < 10.6%). In the GWBP, we observed a population $\overline{\mathrm{F} }$ of 0.35, and we deduced a population $\overline{\mathrm{S} }$ of 0.52 [Eq. (4)].

The 50-marker F estimates (Fig. 4b) yielded identical values for both parameters ($\overline{\mathrm{F} }$=0.35; $\overline{\mathrm{S} }$=0.52). Among the 624 individuals, 34 were classified as highly inbred individuals (5.4% with F_L_ ≥ $0.8\overline{3 }$). Through Axiom chip genotyping 31 of them (with 11,467 SNP markers), we revealed that 26 were truly highly inbred individuals (84%), while five were found to be insufficiently inbred individuals.

In the two generations of the synthetic cultivar from NPZ (Fig. 4c and d), we found a population $\overline{\mathrm{F} }$ of 0.49 in Syn-4 and 0.37 in Syn-8, and deduced a population $\overline{\mathrm{S} }$ of 0.65 and 0.54, respectively [Eq. (5)]. The number of highly inbred individuals (F_L_ ≥ $0.8\overline{3 }$) was 70 (15.4%) in Syn-4 and 46 (13.3%) in Syn-8.

### Classification of highly inbred individuals

**Fig. 5:**
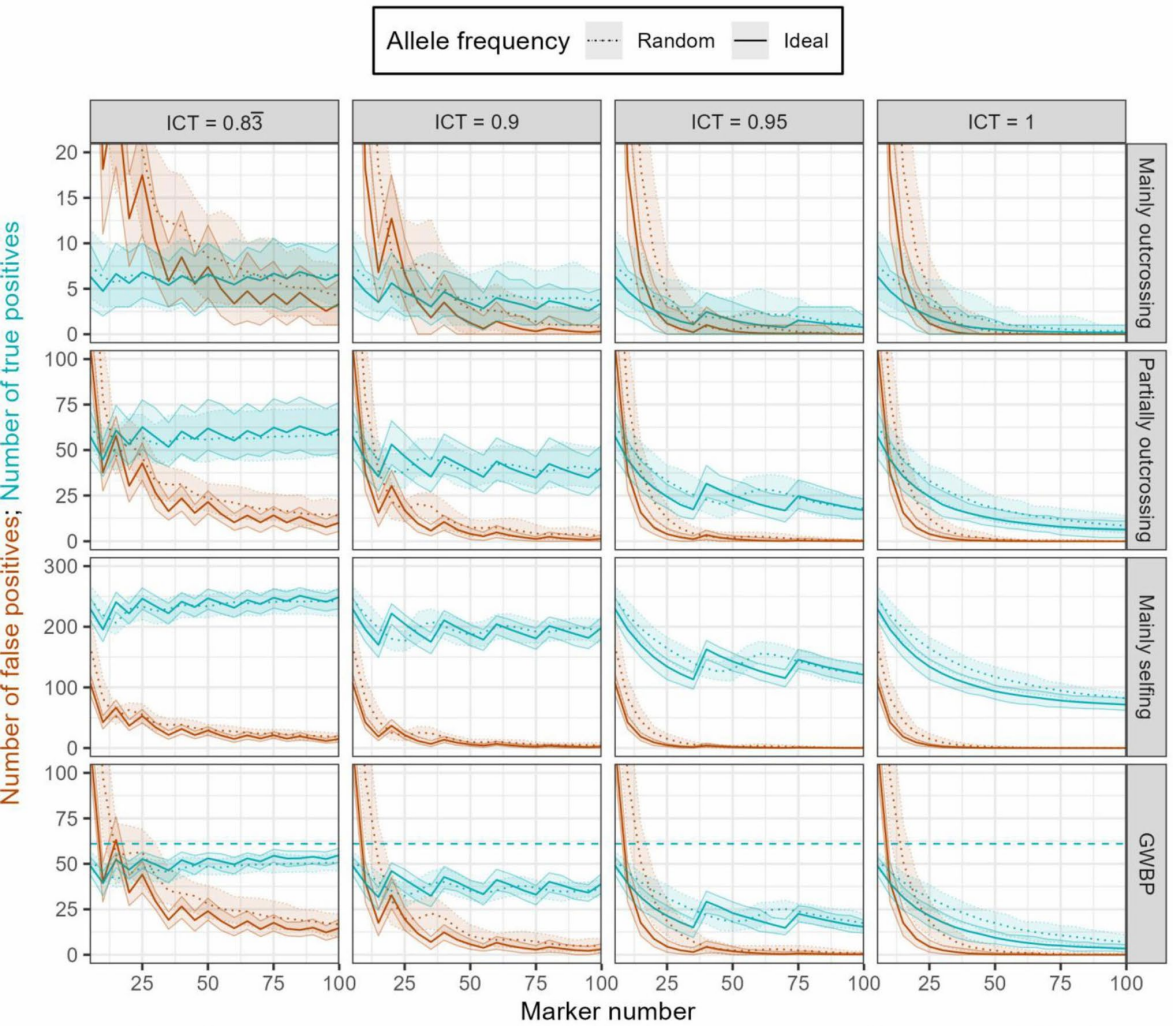
Number of true positives and false positives (among 728 individuals) resulting from the classification of highly inbred individuals according to the number of markers used for F estimation, their allele frequency (linetype), the inbreeding classification threshold used (ICT; vertical facets), in our three simulated populations (mainly outcrossing, partially outcrossing and mainly selfing) and the GWBP (horizontal facets). Note that the y-axis scale varies depending on the number of total positives available in the population. For the GWBP results, we indicated the number of total positives (61), i.e. the maximum number of TP, by a horizontal blue dashed line. The lines represent the mean, and the ribbons represent the 90% confidence interval across the 50 markers subsets

The classification performance is highly impacted by the degree of outcrossing of the population (Fig. 5). The lower the outcrossing, the more effective the classification was. For example, using 50 ideal markers with an ICT of $0.8\overline{3 }$, the classification resulted in seven, 62 and 247 TP, and seven, 21 and 29 FP on average, in the mainly outcrossing, partially outcrossing and mainly selfing populations respectively. The ratio of TP over FP was, respectively, three and nine times greater in the partially outcrossing and mainly selfing populations in comparison with the mainly outcrossing population. A greater accuracy for F estimation (i.e. a greater marker number with ideal instead of random allele frequencies) and a greater ICT always decreased the number of FP. This was not true for the number of TP. When the chosen ICT was close to the inbreeding threshold (F = $0.8\overline{3 }$), a higher accuracy for F estimation increased the number of TP, since it decreased the number of highly inbred individuals wrongly classified as insufficiently inbred (false negatives). In contrast, when the chosen ICT was close to 1, a lower accuracy for F estimation increased the number of TP, since truly highly inbred individuals with a true F value lower than 1 may have been estimated to be fully inbred (F = 1).

Results obtained from the simulated partially outcrossing population are sensibly similar to those observed with the GWBP (Fig. 5), demonstrating good transferability of the simulated results to an existing population. Simulations allowed robust predictions of the number of TP and FP in the GWBP. Assuming as example that we have an ideal marker subset of 50 markers, with ICT = 0.9, the classification resulted in 39 TP and four FP on average in simulations, and there were 36 TP and six FP in our real population.

Classifying highly inbred individuals with a limited number of markers always comes with the cost of including some insufficiently inbred ones. In the GWBP, the only configuration allowing zero FP, was using at least 80 ideal markers and ICT = 1. Then, the classification was not effective, resulting in maximum eight TP. For an effective classification, the appropriate balance should be found between the number of markers and the chosen ICT, considering the expected population composition.

### The preliminary classification step

**Fig. 6:**
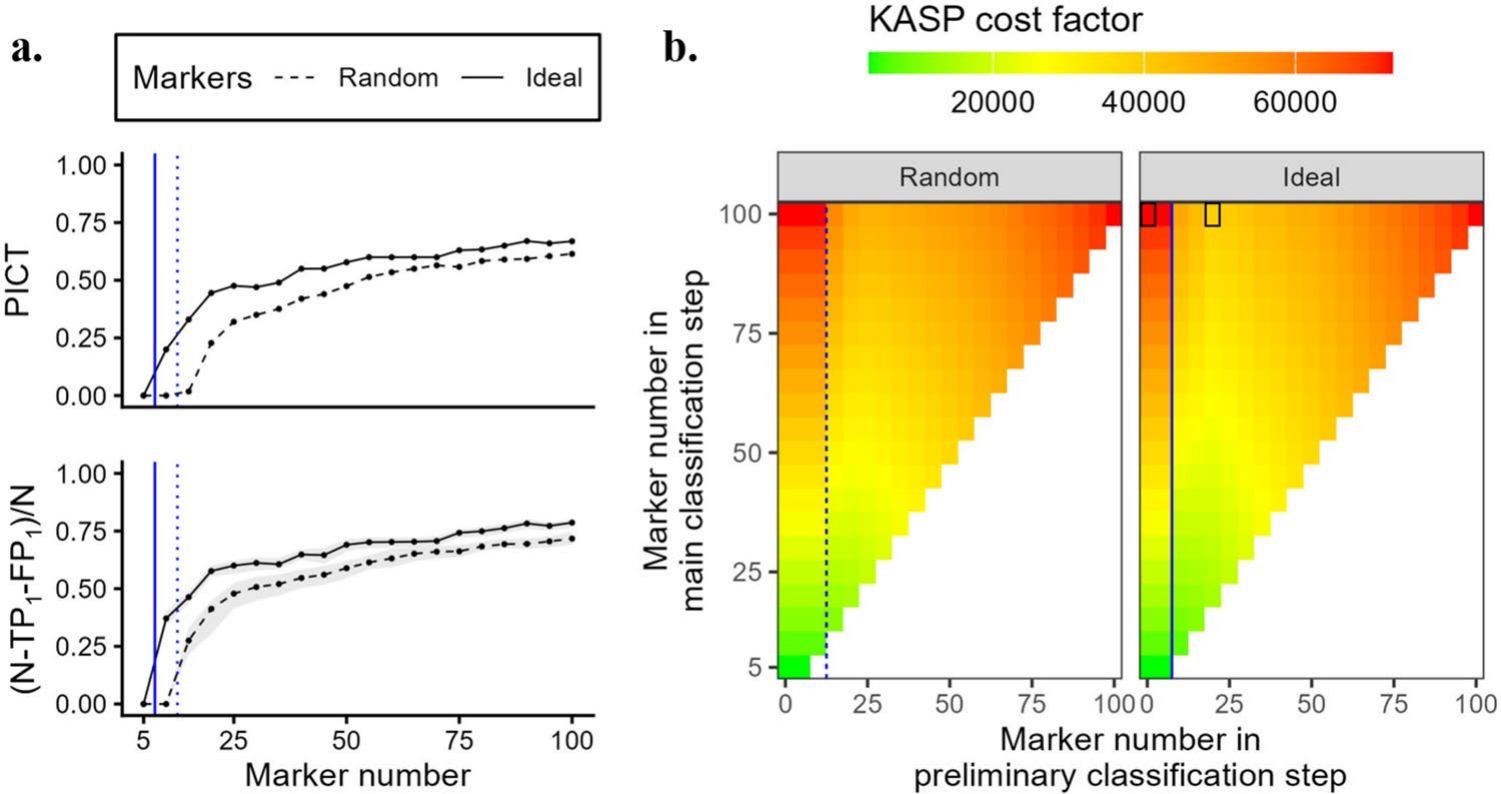
**a** The values of preliminary inbreeding classification threshold (PICT) and the proportion of individuals classified as insufficiently inbred individuals and eliminated in the preliminary classification step [(N—TP_1_—FP_1_) / N] according to the number of markers used and their allele frequencies (random or ideal; linetype). The black lines represent the mean and the ribbons represent the range across the 50 markers subsets. **b** Genotyping cost expressed as a multiplier to the cost of KASP analysis per individual per marker (KASP cost factor), according to the number of markers used in the preliminary and main classification steps, and the type of marker subset (random or ideal). The blue vertical lines show the minimum number of random (dotted line) and ideal (solid line) markers to perform the preliminary classification. See text for the cells highlighted by black boxes

Applying the PICT from simulations to the preliminary classification step in empirical data allowed elimination of many insufficiently inbred individuals (Fig. 6a), while classifying all highly inbred individuals in a vast majority of cases. Only a few marker subsets used for F estimation led to one of the 61 available highly inbred individuals to be excluded. A minimum of respectively 15 and 10 markers were needed with random and ideal markers, to allow the preliminary classification at all (i.e. to obtain a PICT greater than 0; see blue vertical lines in Fig. 6). Starting from this marker number, the marker number in the preliminary classification step displayed a positive relationship with the number of individuals classified as insufficiently inbred (Fig. 6a). Using ideal markers, from 37% (with 10 markers) to 79% (with 100 markers) of the individuals of the GWBP could be eliminated on average using this preliminary classification step.

The cost of the approach without using the preliminary classification step is shown in Fig. 6b in case of using zero marker in the preliminary classification step. Indeed, using the preliminary classification allowed to significantly reduce genotyping cost. Greatest saving was obtained with a low number of markers (from 15 to 30 markers). A greater number of individuals eliminated in the preliminary classification step (Fig. 6a) by a greater number of markers could not compensate their greater genotyping cost (Fig. 6b). For example, with our population of 728 individuals, we could save up to 46% of genotyping cost, in case of using 20 markers in the preliminary classification step (M_1_ = 20) and 80 markers more for the main classification step (M_2_ = 20 + 80 = 100; see the two cells highlighted as black boxes in Fig. 6b).

In addition, we deduced PICT values for the simulated mainly outcrossing and mainly selfing populations. The mainly outcrossing population had greater PICT values than the partially outcrossing population, while those of the mainly selfing population were slightly lower. For example, using 20 ideal markers, the mainly outcrossing, partially outcrossing and mainly selfing populations had a PICT value of respectively 0.50, 0.45 and 0.40.

The classification of highly inbred individuals can be made cheap by the use of a limited number of markers. It can be made even cheaper by including a preliminary classification step with fewer markers, without compromising performance.

## Discussion

A significant proportion of highly inbred individuals was present in the Göttingen Winter Bean Population (GWBP). These individuals can be identified by estimating their individual F values even with only a limited number of markers (e.g. via the likelihood estimator F_L_). Highly inbred individuals can be effectively classified using the ICT that specifically matches the marker set used for F estimation and the expected composition of the population under investigation. The classification can be made cheaper by including a preliminary classification step, without compromising performance. Through our results, breeders can define an appropriate number of markers to cost-efficiently identify individuals from their own faba bean population.

### Inbreeding coefficient estimation

To improve the cost efficiency of the approach, we decided to use only a limited number of KASP markers, instead of the costly high-density genotyping techniques available in faba bean. The cost efficiency of the latter techniques depends, among other factors, largely on genotype sample size, which is probably rather small in case of faba bean. Breeding programmes of minor crops like faba bean are typically smaller in scale compared to those of major crops, discouraging the intensive use of such techniques. We are convinced that for the practical implementation of the approach in faba bean breeding, it was crucial to focus on a cheap approach; yet, future developments may alter these considerations.

With ideal markers, we recommend using the Eq. (2) to estimate individual F values. When it comes to using markers with different allele frequencies (random), we recommend using the likelihood estimator F_L_, although its calculation is relatively complex. If simplicity is important, the estimator F_LH1_ (Li & Horvitz 1953; Table 1) simply using the expected heterozygosity of the individual under Hardy–Weinberg equilibrium given the marker allele frequencies may be recommended, since it ranked second in accuracy (Fig. 3a).

### Classification of highly inbred individuals

The number of TP was slightly greater with simulated data compared to empirical data. The differences for number of TP and FP may be attributed to the difference between the simulated distribution of F values and the real distribution of F values observed in the GWBP. Also, there is zero LD and zero missing value for F estimation in the simulated but certainly not in the empirical setup. None of the F estimators tested in our study integrated algebra to account for LD ≠ 0 between markers. Such LD between the markers used for F estimation reduces the accuracy of F estimates and thus the performance of the classification of highly inbred individuals. Two markers in maximum LD (as extreme assumption) are only as informative on the inbreeding status of an individual as one of the two markers; such LD reduces the effective number of markers.

The sawtooth pattern when using ideal markers (Fig. 5) is caused by the limited resolution of scale for F estimates. The more markers are used to estimate the F of individuals, the higher is the resolution of F. In case of ideal markers, all loci are equally informative and with N markers, the number of possible discrete estimated F values is ⌊(N + 1)/2⌋ + 1, ranging from 0 to 1. With random markers, this pattern was not observed, because there were many more possible values for F estimates, due to varying allele frequency within the marker subset.

### Applications in breeding

To apply the approach, breeders should decide what share of FP among the classified individuals is acceptable, depending on the impact of classifying a truly insufficiently inbred individual as highly inbred for breeding. If the true inbreeding status of the line derived from the individual can be still verified later on, then even a relatively high number of FP may be acceptable. This latter assessment may be achieved by high-throughput genotyping of the identified individuals. The inbreeding status of the individual may also be scrutinized by visual observation of the homogeneity of the derived line in the field. Also, exceeding the defined inbreeding threshold (F = $0.8\overline{3 }$) does not ensure compliance with DUS rules (distinctness, uniformity, stability), as homogeneity for the traits that are used in DUS monitoring is not guaranteed even if a line is derived from an individual with F ≥ $0.8\overline{3 }$. Phenotypic purification and maintenance breeding may be necessary to fix such issues.

After defining the acceptable number of FP, breeders can choose the appropriate ICT based on our simulation results, considering the marker set available for their budget. The choice of the ICT requires some prior information on the degree of outcrossing (1-$\overline{\mathrm{S} }$) of the population of interest to deduce its composition. An estimate of the population $\overline{\mathrm{S} }$ can be deduced from an estimate of the population $\overline{\mathrm{F} }$ via the estimated F values. For a population in equilibrium, $\overline{\mathrm{S} }$ should be deduced from Eq. (4), whereas for a population which did not reach equilibrium, it should be deduced from Eq. (5). Even data acquired with a low number of markers from the preliminary classification step can provide an approximate $\overline{\mathrm{S} }$ estimate and assist decision for the number of markers and the ICT to be used for the main classification step. For example, the $\overline{\mathrm{F} }$ and $\overline{\mathrm{S} }$ estimates of the GWBP based on the 50-marker F estimates (Fig. 4b) demonstrated that a robust estimate can be obtained even when individual F values are estimated from a limited number of markers.

As first rule of thumb, we recommend the breeders to estimate F values of the individuals of their population with 20 near-to-ideal markers and eliminate individuals with a F estimate lower than 0.45. The remaining individuals should be analysed with 30 additional ideal markers for the main classification. Based on results from our GWBP, 42% of individuals of the population should remain after the preliminary classification step, allowing to save about 25% of genotyping costs. In the main classification step, 10.6% should be classified as highly inbred (ICT = $0.8\overline{3 }$), from which 7.3% are TP and 3.3% are FP. Among the 10.6%, we expect only 3.4% of individuals to have an estimated F value greater or equal to 0.95, from which only 0.2% are expected to be FP. These expected results are of course population-specific and are expected to vary with the outcrossing rate of the population, the self-fertilization rate of the inbred lines used as components, and the environmental conditions under which the population was grown.

#### Implications of the use of the approach over successive generations

Will a removal of highly inbred individuals in this generation affect the emergence of highly inbred individuals in the next generation? Probably not, as it is to be expected that populations will produce new, highly inbred individuals in each generation, and because some of the individuals that were previously considered insufficiently inbred will have self-fertilized again by then. The allele frequency of the genes responsible for agronomic traits within the population will stay rather unchanged because the identification, selection and removal of the highly inbred individuals is based on inbreeding and not on a specific trait’s value.

#### Breeding methodology

Given the partial allogamous reproductive mode of faba bean, highly inbred individuals should exist in any faba bean population. A population can be created purposely to let it develop future, new highly inbred genotypes, but they can be identified from now existing populations as well. What matters is that the population to be screened had enough successive generations to produce them. Creating a new population and selecting highly inbred individuals from it is not faster than the conventional process of line breeding. The work load for isolation from pollinators and manual tripping in the conventional approach is avoided and substituted by the investment to identify the naturally occurring highly inbred individuals. However, once this “pipeline” has been set in motion and is running, it yields new inbred individuals in each generation, essentially instantaneous.

In populations initiated from inbred components, F4 individuals can be earliest identified after the fourth generation of such faba bean population. However, given a low outcrossing rate, selection should be postponed to later generations, as the population may contain a significant proportion of genetically unchanged descendants of the inbred components, maintained through selfing. These inbreds are not novel hence uninteresting. For example, we do not recommend applying our approach in the Syn-4 of the synthetic cultivar from NPZ (Fig. 4c), as its four inbred components are expected to account for approximately 8% of the population. Their frequency drops to around 1% in Syn-8 (Fig. 4d).

Our approach can be used as a second-cycle breeding strategy. Already existing promising populations can be exploited to provide novel, highly inbred, superior individuals directly and thus grant a tremendous gain of time for breeding.

Our approach can be implemented in the two-part breeding strategy described by Adhikari et al. (2021) for faba bean breeding, a revised version of the strategy presented by Gaynor et al. (2017). Combined with genomic prediction for agronomic value, individuals from a fast-cycling population can be selected for both their F and their genomic estimated breeding value (GEBV) of pertinent traits. The selected elite inbred individuals would enter the cultivar production component. Phenotypic data collected in multilocation yield trials during the cultivar production can be used to re-train a genomic prediction model.

Insufficiently inbred individuals with high GEBV can also be selected from faba bean populations. They can be used for line breeding (beware problems with DUS), or as a component for a synthetic cultivar. If used as component, their dose in Syn-0 to initiate the synthetic cultivar should be adjusted depending on their F value. One zero-inbred genotype is on the scale of population size, equivalent to two fully inbred lines.

Even without genomic prediction, the approach can at least be coupled with marker-assisted selection for monogenic or oligogenic traits. By choosing markers for F estimation that are known to be significantly associated with major QTLs, highly inbred individuals can be identified and simultaneously judged for such traits. As an example, for faba bean, the KASP markers developed for vicine–convicine (VC) content (Khazaei et al. 2017; Ugwuanyi et al. 2025) could be added in the F estimation and allow prediction of VC (if there is such diversity) without extra effort.

In this study, we focused on faba bean populations, but our approach is potentially applicable to any partially allogamous crop, such as rapeseed (*Brassica napus*; S ∈ [0.53, 0.88]; Becker et al. 1992), sorghum (*Sorghum bicolor* (L.) Moench; S ∈ [0.70, 0.99]; Barnaud et al. 2008), pigeon pea (*Cajanus cajan* (L.) Millsp.; S ∈ [0.3, 0.8]; Saxena et al. 2013), alfalfa (*Medicago sativa*; S ∈ [0.05, 0.30]; Dieterich Mabin et al. 2021), peppers (*Capsicum annuum* L.; S ∈ [0.54, 0.99]; Franceschetti 1971), bitter fennel (*Foeniculum vulgare* var. *Vulgare*; S ∈ [0.04, 0,20]; Bahmani et al. 2023) or safflower (*Carthamus tinctorius* L.; S ∈ [0.60, 0.95]; Kumari & Pandey 2005). The only requirement is that the balance between self- and cross-fertilization rate offers enough highly inbred individuals and genetic recombinations.

The described approach makes it possible to realize an old dream, namely to directly exploit the natural crossings and selfings in faba bean populations as cheap substitute for the first phase of conventional line breeding, namely the procuring of genetic variation (Schnell 1982). An appropriate implementation in modern faba bean breeding requires further analysis and ideas.

## Conclusions

Based on the literature and our current findings, we are confident that with a reasonable number of markers, highly inbred individuals from faba bean populations of temperate agroecosystems can be identified with a low number of false discoveries. The use of a preliminary classification step with a very low number of markers will further limit genotyping cost (up to 46%). Preliminary F estimates inform on the presumed outcrossing rate of the population, allowing optimization of the number of markers used and the F value to use as classification threshold for the main classification step, depending on the desired classification performance.

Faba bean breeding can greatly benefit from this approach, allowing fast, cheap and effortless access to faba bean inbred lines, in comparison with the conventionally laborious process of inbred line production, as an alternative to the non-available DH approach.

## Supplementary Information

Below is the link to the electronic supplementary material.

Supplementary file1 (DOCX 180 kb)

Supplementary file2 (DOCX 18 kb)

Supplementary file3 (DOCX 491 kb)

Supplementary file4 (XLSX 22 kb)

## Data Availability

The data sets employed in this study are available from the corresponding author on reasonable request.

## Code availability

The R code scripts used for the analyses are available from the corresponding author on reasonable request.
