# Supplementary material for: Faba bean populations already contain the inbreds needed for breeding
Source: Theor Appl Genet. 2026 Jun 12;139(7):178. doi: 10.1007/s00122-026-05259-w (PMC13263235; doi:10.1007/s00122-026-05259-w)
Supplement: Supplementary file 1 — Supplementary file1 (DOCX 180 kb) [file 122_2026_5259_MOESM1_ESM.docx]

**Simulation of the theoretical distribution of individual F values within a faba bean population**

Simulations were all done manually in R, without relying on existing packages. First, we calculated the theoretical proportion of individuals for each inbreeding cohort at equilibrium (Fig. 1). Then, considering the existing true quantitative variation in F values within each inbreeding cohort (see equation 1 of the main text with N=24), we calculated for each inbreeding cohort the proportion of individuals per value of F and multiplied it with the proportion of the inbreeding cohort at equilibrium, to obtain the final distribution (Fig. 2).

To calculate the theoretical proportion of individuals for each inbreeding cohort at equilibrium, we considered both the self-fertilization rate (S) and the seed number per plant of the individuals of a cohort to be dependent on the mean F of the corresponding cohort. In faba bean, non-inbred individuals tend to self-fertilize more and produce more seeds than inbred individuals (Brünjes and Link 2021; Link 1990).

We inspected literature to define realistic values for (1) the S of fully inbred individuals (S_F∞_), (2) the S of non-inbred individuals (S_F1_) and (3) the heterosis for seed number per plant. The degree of outcrossing is highly variable in faba bean, ranging from 10 to 70%, depending on the genotypes composing the population and its growing environment (heat stress, types of pollinator present; Link 1990; Adhikari et al. 2021). Brünjes and Link (2021) reported a S_F∞_ of 53%, lower than that found for S_F1_ (74%). The difference amounted to ~20%. Earlier, Link (1990) reported for spring faba beans S_F∞_ = 35% and S_F1_ = 55%; again, the difference amounted to ~20%. For our simulation, we decided to fix this heterotic difference between S_F∞_ and S_F1_ to 20%. For intermediate cohorts, S resulted from a linear relationship between F and S. Midparent yield heterosis was reported to be 50 to 75% in faba bean (Link 1990; Link et al. 1994); a large share due to heterosis for seed number. We decided to set the heterosis for seed number per plant to 50% and again assume a linear relationship between an individual’s seed number and the mean F of its inbreeding cohort.

We defined in the main text, three equilibrium populations with differing degree of outcrossing: (1) the mainly outcrossing population (S_F∞_ = 10% and S_F1_ = 30%), (2) the partially outcrossing population (S_F∞_ = 40% and S_F1_ = 60%) and (3) the mainly selfing population (S_F∞_ = 70% and S_F1_ = 90%). Their respective distribution of individual F values is shown in the Fig. 2 of the main text.


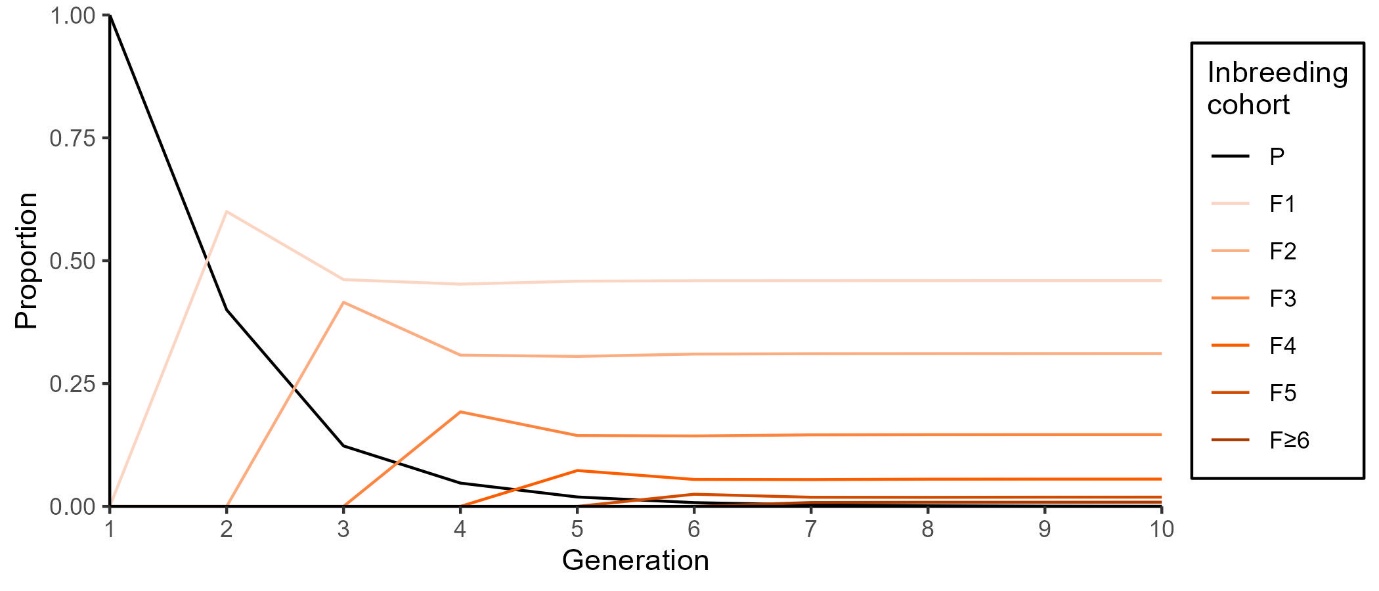


**Fig. 1** Evolution of proportion of inbreeding cohorts within the simulated partially outcrossing population (S_F∞_ = 40% and S_F1_ = 60%) in its first ten generations


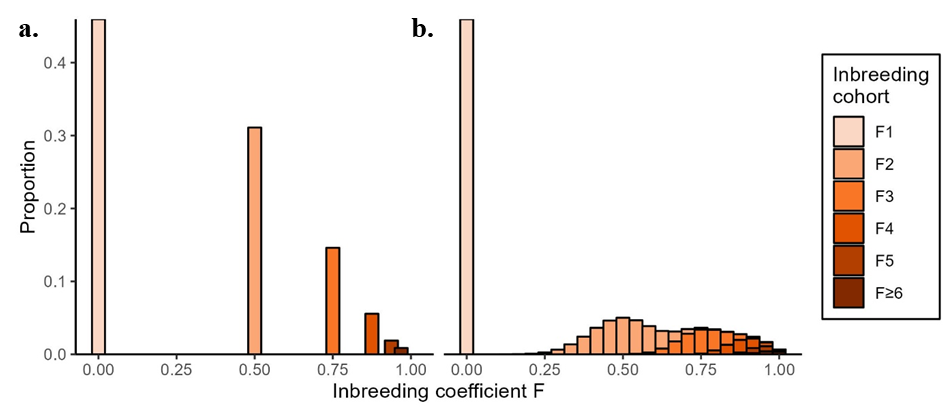


**Fig. 2** Distribution of individual F values within the simulated partially outcrossing population (S_F∞_ = 40% and S_F1_ = 60%) at equilibrium **a** before and **b** after considering the true quantitative variation of F values within each inbreeding cohort

We note that there was no variation in F values within the F1 cohort (Fig. 2b), because we considered each individual to have an infinite number of loci. The variation of F values in F≥2 cohorts originates from the independently segregating genomic segments (N=24).

With the unreal setting of zero variation of F values per inbreeding cohort (Fig. 2a), all individuals belonging to the cohort F1, F2 or F3 were defined as insufficiently inbred (F<$0.8\bar{3}$), while those belonging to the F≥4 cohorts were defined highly inbred (F≥$0.8\bar{3}$). When implementing the variation of F values per inbreeding cohort (Fig. 2b), some F2 and F3 individuals were defined as highly inbred, and some F4 individuals were defined as insufficiently inbred. In case of the simulated partially outcrossing population (S_F∞_ = 40% and S_F1_ = 60%), 0.61% of the population was insufficiently inbred F4 individuals (F<$0.8\bar{3}$), while 2.86% was highly inbred F2 or F3 individuals (F≥$0.8\bar{3}$). Hence, the number of individuals defined as highly inbred was found to be slightly greater when the variation of F values per inbreeding cohort was considered.

The framework to generate the distribution of individual F values from an idealized faba bean population in our study follows several simplifying assumptions. It is deterministic and does not fully reflect the complexity underlying the actual genetic composition of real populations, neglecting for example non-heterotic genetic variation of self-fertilization rate.

**References**

Adhikari, K. N., Khazaei, H., Ghaouti, L., Maalouf, F., Vandenberg, A., Link, W., & O’Sullivan, D. M. (2021). Conventional and Molecular Breeding Tools for Accelerating Genetic Gain in Faba Bean (*Vicia faba* L.). Frontiers in Plant Science, 12, 744259. https://doi.org/10.3389/fpls.2021.744259

Brünjes, L., & Link, W. (2021). Paternal outcrossing success differs among faba bean genotypes and impacts breeding of synthetic cultivars. Theoretical and Applied Genetics, 134(8), 2411–2427. https://doi.org/10.1007/s00122-021-03832-z

Link, W. (1990). Autofertility and rate of cross-fertilization: Crucial characters for breeding synthetic varieties in faba beans (*Vicia faba* L.). Theoretical and Applied Genetics, 79(5), 713–717. https://doi.org/10.1007/BF00226888

Link, W., Ederer, W., & von Kittlitz, E. (1994). Zuchtmethodische Entwicklungen: Nutzung von Heterosis bei Fababohnen. Vorträge für Pflanzenzüchtung, 30, 201–230.
