# Supplementary material for: Faba bean populations already contain the inbreds needed for breeding
Source: Theor Appl Genet. 2026 Jun 12;139(7):178. doi: 10.1007/s00122-026-05259-w (PMC13263235; doi:10.1007/s00122-026-05259-w)
Supplement: Supplementary file 2 — Supplementary file2 (DOCX 18 kb) [file 122_2026_5259_MOESM2_ESM.docx]

| **Estimator** | **Formula** | **Formula in reference paper** | **References** |
| --- | --- | --- | --- |
| **F_LH1_** | $F_{LH1}=1- \frac{\sum_{i=1}^{m} x_{i}(2-x_{i})}{\sum_{i=1}^{m} 2p_{i}(1-p_{i})}$  where, m is the total number of markers, x_i_ the number of minor alleles at locus i, and p_i_ the major allele frequency at locus i. | $f=1- \frac{H}{H_{0}}$  where, H is the number of observed heterozygous loci, and H_0_ the number of expected heterozygous loci assuming Hardy-Weinberg equilibrium, calculated as the sum of expected probability of being heterozygous of all loci, based on their allele frequencies. | Li & Horvitz 1953 (also described by Ritland 1996 as a second simple method-of-moments estimator). |
| **F_LH2_** | $F_{LH2}=1-\frac{1}{m}\sum_{i=1}^{m} \frac{x_{i}(2-x_{i})}{2p_{i}(1-p_{i})}$ | $F_{LH2}=1- \frac{\sum_{k=1}^{S} \frac{x_{k}(2- x_{k})}{2p_{k}(1-p_{k})}}{S}$  where, S is the total number of loci (k = 1, 2, …, S), x_k_ the number of minor alleles at locus k, and p_k_ the major allele frequency of locus k. | Caballero et al. 2022 (equivalent to the estimator used by Carothers et al. 2006 without considering the weighing of loci by the inverse of their variance). |
| **F_VR1_** | $F_{VR1}=\frac{\sum_{i=1}^{m} {(x_{i}-2p_{i})}^{2}}{\sum_{i=1}^{m} 2p_{i}(1-p_{i})}-1$ | $F= G_{jj}-1$  where,  G = the genomic relationship matrix (GRM).  $G= \frac{(M-P)(M-P)'}{2\sum p_{i}(1-p_{i})}$  where, M is a matrix of dimensions n × m (n = number of individuals; m = number of loci) specifying which marker alleles each individual inherited, P a matrix of order n × m where each element of a column i is equal to 2(p_i_ – 0.5), and p_i_ the major allele frequency of locus i. | VanRaden 2008 |
| **F_VR2_** | $F_{VR2}=\frac{1}{m}\sum_{i=1}^{m} \left( \frac{{(x_{i}-2p_{i})}^{2}}{2p_{i}(1-p_{i})}-1 \right)$ | $F= G_{jj}-1$  where, G is the genomic relationship matrix (GRM).  $G=(M-P)D(M-P)'$  where, D is a diagonal matrix with $D_{ii}= \frac{1}{m\left[ 2p_{i}\left( 1-p_{i} \right) \right]}$  where, m is the number of loci, and p_i_ the major allele frequency of locus i. | VanRaden 2008 (using the formula proposed by Leutenegger et al. 2003 and Amin et al. 2007). |
| **F_YA1_** | $F_{YA1}=\frac{\sum_{i=1}^{m} {x_{i}}^{2}-\left( 1+2p_{i} \right)x_{i}+{{2p}_{i}}^{2}}{\sum_{i=1}^{m} 2p_{i}(1-p_{i})}$ | ${\tilde{f}_{{UNI}_{j}}}^{w}= \frac{\sum_{l=1}^{L} [{X_{jl}}^{2}-\left( 1+2\tilde{p}_{l} \right)X_{jl}+2{\tilde{p}_{l}}^{2}]}{\sum_{l=1}^{L} {2\tilde{p}}_{l}(1-\tilde{p}_{l})}$  where, L is the total number of loci, X_jl_ the number of copies major allele of individual j at locus l, and $\tilde{p}_{l}$ the sample allele proportion of locus l. | Zhang et al. 2022 (based on Yang et al. 2010) |
| **F_YA2_** | $F_{YA2}=\frac{1}{m}\sum_{i=1}^{m} \frac{{x_{i}}^{2}-\left( 1+2p_{i} \right)x_{i}+{{2p}_{i}}^{2}}{2p_{i}(1-p_{i})}$ | $F= \prod_{i=1}^{N} \left( A_{ijj}-1 \right)$  where, A is the genomic relationship matrix, and N the total number of loci.  $A=\frac{(M-P)(M-P)'}{N}$  where, M is a matrix of dimensions n × m (n = number of individuals; m = number of loci) specifying which marker alleles each individual inherited, and P a matrix of order n × m where each element of a column i is equal to 2(p_i_ – 0.5).  $A_{ijj}=1+\frac{x_{ij}-\left( 1+2p_{i} \right)x_{ij}+2{p_{i}}^{2}}{2p_{i}(1-p_{i})}$  where, x_ij_ is the number of copies major allele of individual j at locus i, and p_i_ the major allele frequency of locus i. | Yang et al. 2010 |

**References**

Amin, N., Duijn, C. M. van, & Aulchenko, Y. S. (2007). A Genomic Background Based Method for Association Analysis in Related Individuals. PLOS ONE, 2(12), e1274. https://doi.org/10.1371/journal.pone.0001274

Caballero, A., Fernández, A., Villanueva, B., & Toro, M. A. (2022). A comparison of marker-based estimators of inbreeding and inbreeding depression. Genetics Selection Evolution, 54(1), 82. https://doi.org/10.1186/s12711-022-00772-0

Carothers, A. D., Rudan, I., Kolcic, I., Polasek, O., Hayward, C., Wright, A. F., Campbell, H., Teague, P., Hastie, N. D., & Weber, J. L. (2006). Estimating Human Inbreeding Coefficients: Comparison of Genealogical and Marker Heterozygosity Approaches. Annals of Human Genetics, 70(5), 666–676. https://doi.org/10.1111/j.1469-1809.2006.00263.x

Leutenegger, A.-L., Prum, B., Génin, E., Verny, C., Lemainque, A., Clerget-Darpoux, F., & Thompson, E. A. (2003). Estimation of the Inbreeding Coefficient through Use of Genomic Data. The American Journal of Human Genetics, 73(3), 516–523. https://doi.org/10.1086/378207

Li, C. C., & Horvitz, D. G. (1953). Some methods of estimating the inbreeding coefficient. American Journal of Human Genetics, 5(2), 107–117.

Ritland, K. (1996). Estimators for pairwise relatedness and individual inbreeding coefficients. Genetics Research, 67(2). https://www.cambridge.org/core/journals/genetics-research/article/estimators-for-pairwise-relatedness-and-individual-inbreeding-coefficients/9AE218BF6BF09CCCE18121AA63561CF7

VanRaden, P. M. (2008). Efficient Methods to Compute Genomic Predictions. Journal of Dairy Science, 91(11), 4414–4423. https://doi.org/10.3168/jds.2007-0980

Yang, J., Benyamin, B., McEvoy, B. P., Gordon, S., Henders, A. K., Nyholt, D. R., Madden, P. A., Heath, A. C., Martin, N. G., Montgomery, G. W., Goddard, M. E., & Visscher, P. M. (2010). Common SNPs explain a large proportion of the heritability for human height. Nature Genetics, 42(7), 565–569. https://doi.org/10.1038/ng.608

Zhang, Q. S., Goudet, J., & Weir, B. S. (2022). Rank-invariant estimation of inbreeding coefficients. Heredity, 128(1), 1–10. https://doi.org/10.1038/s41437-021-00471-4
