# Supplementary material for: Faba bean populations already contain the inbreds needed for breeding
Source: Theor Appl Genet. 2026 Jun 12;139(7):178. doi: 10.1007/s00122-026-05259-w (PMC13263235; doi:10.1007/s00122-026-05259-w)
Supplement: Supplementary file 3 — Supplementary file3 (DOCX 491 kb) [file 122_2026_5259_MOESM3_ESM.docx]

**The likelihood estimator F_L_**

The so-called likelihood estimator F_L_ is based on Wright's (1921) equilibrium genotypic frequencies for partially allogamous populations. This estimator represents a streamlined alternative to the more complex approaches of Wang (2011) and Hall et al. (2012). Despite its simplicity, we found no prior example of our use of this in literature.

$F_{L}=argmaxl\left( F \right)$, (1)

where l(F) is the joint log-likelihood of observing the combination of genotypes based on the available marker data.

$l(F)= \sum_{i=1}^{m} log(P(g_{i}|F,p_{i}))$, (2)

where m is the total number of markers, g_i_ the observed genotype at locus i, and p_i_ the major allele frequency at locus i.

$P\left( g_{i} | F,p_{i} \right)=\left\{ \begin{aligned} {p_{i}}^{2}+p_{i}\left( 1-p_{i} \right)F , &g_{i}=A_{i}A_{i} \\ 2p_{i}(1-p_{i})(1-F), &g_{i}=A_{i}a_{i} \\ \left( 1-p_{i} \right)^{2}+p_{i}\left( 1-p_{i} \right)F, &g_{i}=a_{i}a_{i} \end{aligned} \right.$, (3)

where A_i_ is the most frequent (major) allele at locus i, and a_i_ the minor allele at locus i.


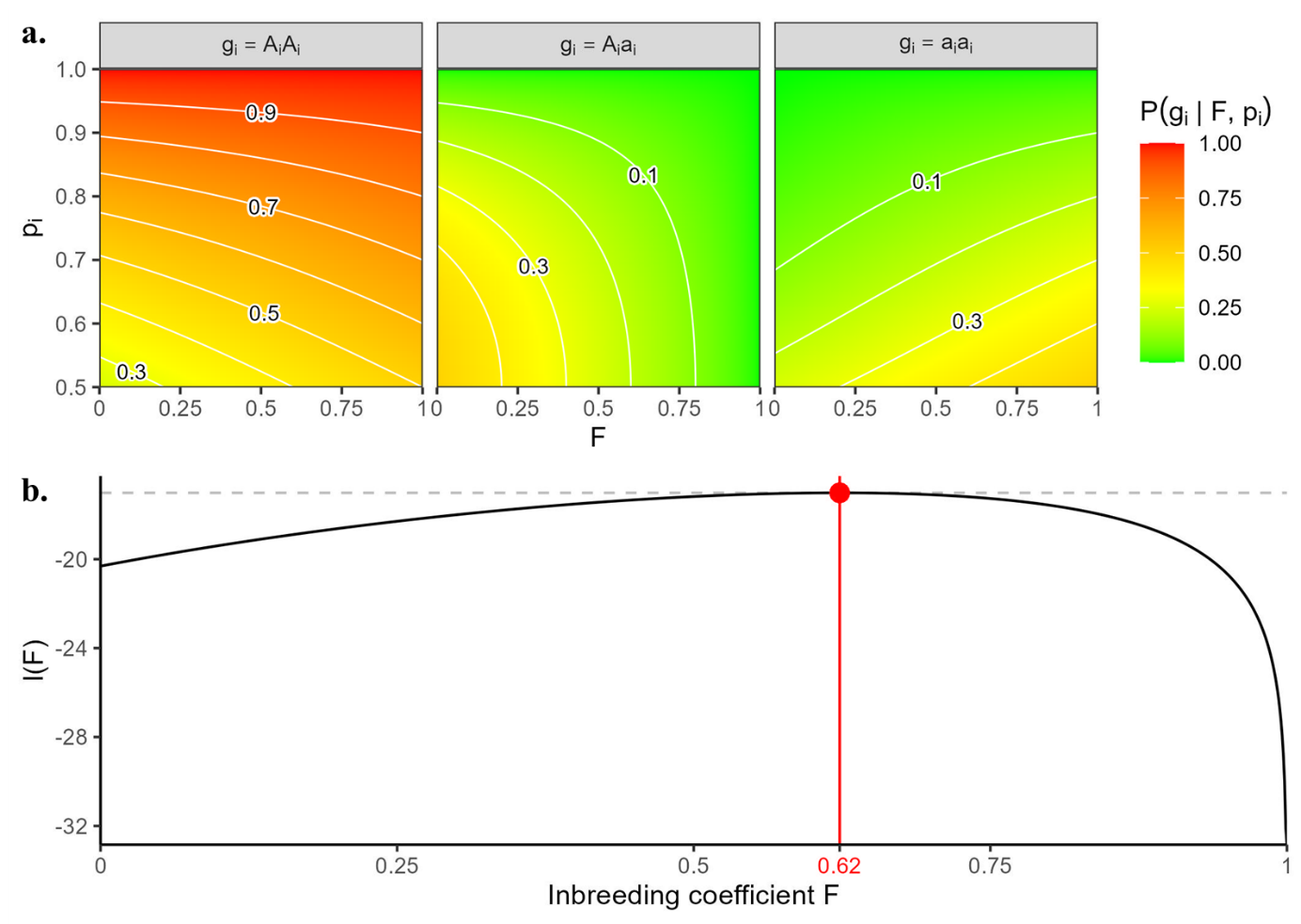


**Fig. 1** Likelihood estimation of an individual’s inbreeding coefficient **a** at one locus: probability of observing the genotype at locus i according to its allele frequency p_i_ and inbreeding coefficient F for each of the three possible genotypes for a biallelic locus. **b** with 25 loci: joint log-likelihood of observing the combination of marker genotypes at 25 random loci for an individual of the GWBP (see the detailed calculation in Supplementary material 4)

P(g_i_│F,p_i_) is the probability of observing a specific genotype g_i_ according to F given the allele frequency of the locus p_i_ (Fig. 1a). This probability forms the likelihood of F for each locus, based on its genotype g_i_ and allele frequency p_i_. For example, if we have a marker with allele frequency p=0.8, a half-inbred individual (F=0.5) will have a probability of 0.72, 0.16 and 0.12, to be respectively homozygous for the major allele, heterozygous and homozygous for the minor allele. The probability of this individual to be homozygous for the major allele if non-inbred (F=0), would only be 0.64.

To sum the log-likelihood across all employed markers, we assume that they are independent (LD=0). In the example given in Fig. 1b, we estimated F of one individual from the GWBP from 25 random markers. From a homozygosity rate of 0.88, we obtained a likelihood estimate of 0.62 using the estimator F_L_. The ratio of likelihood of two F values can be obtained by exponentiating the difference between their respective l(F). For example, between F=0.62 (l(F)=-17.01; the value with highest l(F)) and F=0 (l(F)=-20.31), there is a difference of l(F) of 3.29, meaning that the individual is around 27 times more likely to have a F of 0.62 than 0 (Azzalini, 1996). This result should be interpreted with care considering the strong assumption that there is zero LD between the loci.

**Reference**

Azzalini, A. (1996). Statistical Inference—Based on the likelihood (Chapman&Hall). https://www.routledge.com/Statistical-Inference-Based-on-the-likelihood/Azzalini/p/book/9781032478012?srsltid=AfmBOoqucy5ms7K7ulpz2XOcVIBMqpOK4E28_F6gcyGtO3rAAH8Vir63

Hall, N., Mercer, L., Phillips, D., Shaw, J., & Anderson, A. D. (2012). Maximum likelihood estimation of individual inbreeding coefficients and null allele frequencies. Genetics Research, 94(3), 151–161. https://doi.org/10.1017/S0016672312000341

Wang, J. (2011). A new likelihood estimator and its comparison with moment estimators of individual genome-wide diversity. Heredity, 107(5), 433–443. https://doi.org/10.1038/hdy.2011.30

Wright, S. (1921). Systems of Mating. I. The Biometric Relations between Parent and Offspring. Genetics, 6(2), 111–123. https://doi.org/10.1093/genetics/6.2.111
